# Altered Hepatic Lipid Metabolism Contributes to Nonalcoholic Fatty Liver Disease in Leptin-Deficient Ob/Ob Mice

**DOI:** 10.1155/2013/296537

**Published:** 2013-01-16

**Authors:** James W. Perfield, Laura C. Ortinau, R. Taylor Pickering, Meghan L. Ruebel, Grace M. Meers, R. Scott Rector

**Affiliations:** ^1^Department of Nutrition and Exercise Physiology, University of Missouri, Columbia, MO 65211, USA; ^2^Department of Food Science, University of Missouri, Columbia, MO 65211, USA; ^3^Division of Gastroenterology and Hepatology, Department of Internal Medicine, University of Missouri, Columbia, MO 65211, USA; ^4^Harry S. Truman Memorial Veterans' Medical Center, Columbia, MO 65201, USA

## Abstract

Nonalcoholic fatty liver disease (NAFLD) is strongly linked to obesity, insulin resistance, and abnormal hepatic lipid metabolism; however, the precise regulation of these processes remains poorly understood. Here we examined genes and proteins involved in hepatic oxidation and lipogenesis in 14-week-old leptin-deficient Ob/Ob mice, a commonly studied model of obesity and hepatic steatosis. Obese Ob/Ob mice had increased fasting glucose, insulin, and calculated HOMA-IR as compared with lean wild-type (WT) mice. Ob/Ob mice also had greater liver weights, hepatic triglyceride (TG) content, and markers of *de novo* lipogenesis, including increased hepatic gene expression and protein content of acetyl-CoA carboxylase (ACC), fatty acid synthase (FAS), and stearoyl-CoA desaturase-1 (SCD-1), as well as elevated gene expression of PPAR**γ** and SREBP-1c compared with WT mice. While hepatic mRNA levels for PGC-1**α**, PPAR**α**, and TFAM were elevated in Ob/Ob mice, measures of mitochondrial function (**β**-HAD activity and complete (to CO_2_) and total mitochondrial palmitate oxidation) and mitochondrial OXPHOS protein subunits I, III, and V content were significantly reduced compared with WT animals. In summary, reduced hepatic mitochondrial content and function and an upregulation in *de novo* lipogenesis contribute to obesity-associated NAFLD in the leptin-deficient Ob/Ob mouse.

## 1. Introduction

Nonalcoholic fatty liver disease (NAFLD) is the most common cause of chronic liver disease in US adults [1]. NAFLD rates are as high as 75–100% in obese and morbidly obese individuals, and NAFLD is strongly linked to insulin resistance and the metabolic syndrome [2, 3]. Animal models of NAFLD are critical for advancing our understanding of the pathogenesis of NAFLD as well as investigating the therapeutic effects of proposed therapies. A range of animal models have been used for the study of NAFLD [4–6]; however, the translational value of these models is dependent on the extent to which NAFLD has been characterized and is understood in that model. 

We have previously shown that hepatic mitochondrial dysfunction is a significant contributing factor to NAFLD development and progression in the hyperphagic, obese Otsuka Long-Evans Tokushima Fatty (OLETF) rat, a commonly studied model of insulin resistance and type 2 diabetes [7]. Another commonly studied model of hyperphagia-induced obesity and insulin resistance is the leptin-deficient Ob/Ob mouse [8–12]. It has been demonstrated that Ob/Ob mice develop NAFLD similar to that observed in human patients [13]. However, whether NAFLD development in Ob/Ob mice is associated with direct alterations in hepatic lipid metabolism still remains poorly understood. Therefore, in this study, we sought to determine whether NAFLD development was due to both a disruption in hepatic mitochondrial content and function as well as an upregulation of hepatic *de novo* lipogenesis markers in the commonly studied model of obesity, the leptin-deficient Ob/Ob mouse. 

## 2. Materials and Methods

### 2.1. Animals and Animal Care

The University of Missouri Animal Care and Use committee approved all procedures involving the mice. Animals were maintained at a controlled temperature (22°C) and a 12 hr light : 12 hr dark cycle. Five-week-old male Ob/Ob and C57BL/6 (WT) mice were purchased from the Jackson Laboratory, individually housed and fed an AIN-93G diet for 9 wks. Food intake and body weight were measured weekly.

### 2.2. Tissue Collection and Histological Analysis

At 14 wks of age, animals were fasted 10–12 hr, and blood glucose was measured using a handheld glucometer (ReliOn micro). Animals were then euthanized by CO_2_ asphyxiation followed by exsanguination via cardiac puncture. Plasma was separated by centrifugation, aliquoted, and frozen for future analysis. Gonadal adipose tissue was removed and weighed. The liver was removed and weighed and either flash frozen in liquid nitrogen, placed in 10% formalin, or placed in ice-cold buffer (100 Mm KCl, 40 mM Tris-HCl, 10 mM Tris-Base, 5 mM MgCl_2_ *·*6H_2_O, 1 mM EDTA, and 1 mM ATP; pH 7.4) for fresh tissue hepatic fatty acid oxidation assays as previously described [14]. Fixed tissue was embedded in paraffin, sectioned and stained with hematoxylin and eosin (H & E staining) for histological analysis. For all animals, digital images of 3 to 5 fields of view were acquired using an Olympus BX51 light microscope and Olympus DP70 camera.

### 2.3. Fatty Acid Oxidation

Palmitate oxidation was measured with radiolabeled [1-^14^C]palmitate (American Radiochemicals) in fresh liver homogenate preparations as previously reported [14]. Both ^14^CO_2_, representing complete fatty acid oxidation, and ^14^C labeled acid soluble metabolites (ASMs), representing incomplete oxidation, were collected in the previously described trapping device and then counted on a liquid scintillation counter. Palmitate oxidation experiments were performed in the presence (100 uM) or absence of etomoxir (a specific inhibitor of mitochondrial carnitine palmitoyl-CoA transferease-1 and entry into the mitochondria) in order to examine the relative contribution of mitochondrial (−etomoxir) and extra mitochondrial organelles (+etomoxir) in total fatty acid oxidation as previously described [15].

### 2.4. Liver Triglyceride Analysis 

Liver triglyceride content was determined using a modified protocol described by Schwartz and Wolins [16]. Briefly, liver (~30 mg) was homogenized using a TissueLyser (Qiagen), lipid was extracted using chloroform/methanol (1 : 2, v/v) and 4 mM MgCl_2_ solutions. The organic phase was then separated, dried, reconstituted in butanol-Triton X-114 (3 : 2, v/v), and vortexed. Triglyceride was then quantified using a colorimetric enzyme-linked kit (Sigma; St. Louis, MO) and concentration was expressed as nanomoles per gram wet weight [14].

### 2.5. Insulin Sensitivity

Fasting plasma insulin was determined using an ELISA kit (Crystal Chem). The homeostasis model assessment (HOMA) was used as a proxy for insulin sensitivity and calculated using fasting insulin and glucose values (glucose (mg/dL) × insulin (uU/mL))/405.

### 2.6. Western Blotting

 Western blot analyses were performed for the determination of the protein content of acetyl coenzyme A carboxylase (ACC) (Cell Signaling, Beverly, CA), fatty acid synthase (FAS) (Cell signaling), stearoyl CoA desaturase (SCD-1)(Alpha Diagnostic International, San Antonio, TX), and oxidative phosphorylation (OXPHOS) complexes I to V of the electron transport chain (MitoProfile Total OXPHOS Rodent WB Antibody Cocktail; MitoSciences, Eugene, OR), using 30 ug of protein per well as previously described [14, 15, 17]. Total protein staining (0.1% amido-black) for each lane was used to correct for any differences in protein loading or transfer of all band densities. The intensities of the bands and total protein staining were quantified using Quantity One software (Bio-Rad).

### 2.7. Real-Time Quantitative PCR

Relative mRNA expression was determined in hepatic tissue using commercially available primers. Total mRNA was extracted from liver using RNeasy mini kits with on-column DNase digestion (Qiagen). Purity and concentration were determined with a Nanodrop 1000 spectrophotometer (Thermo Scientific). 1 *μ*g of RNA was used to synthesize cDNA with a reverse transcriptase polymerase chain reaction kit (Applied Biosystems) and diluted to 10 ng/*μ*L. Expression of mRNA was determined using SYBR green qRT-PCR on an Applied Biosystems StepOne Plus RT-PCR system.  Table 1 provides the sequences for the primers that were used. Fold difference for gene expression was calculated as 2^−ΔΔCT^ using the endogenous control gene *Cyclophilin b*.

### 2.8. Statistics

 Differences were analyzed by Student's *t*-test with significance set at *P* < 0.05. Values are reported as means ± standard error. 

## 3. Results and Discussion

**Figure 1:**
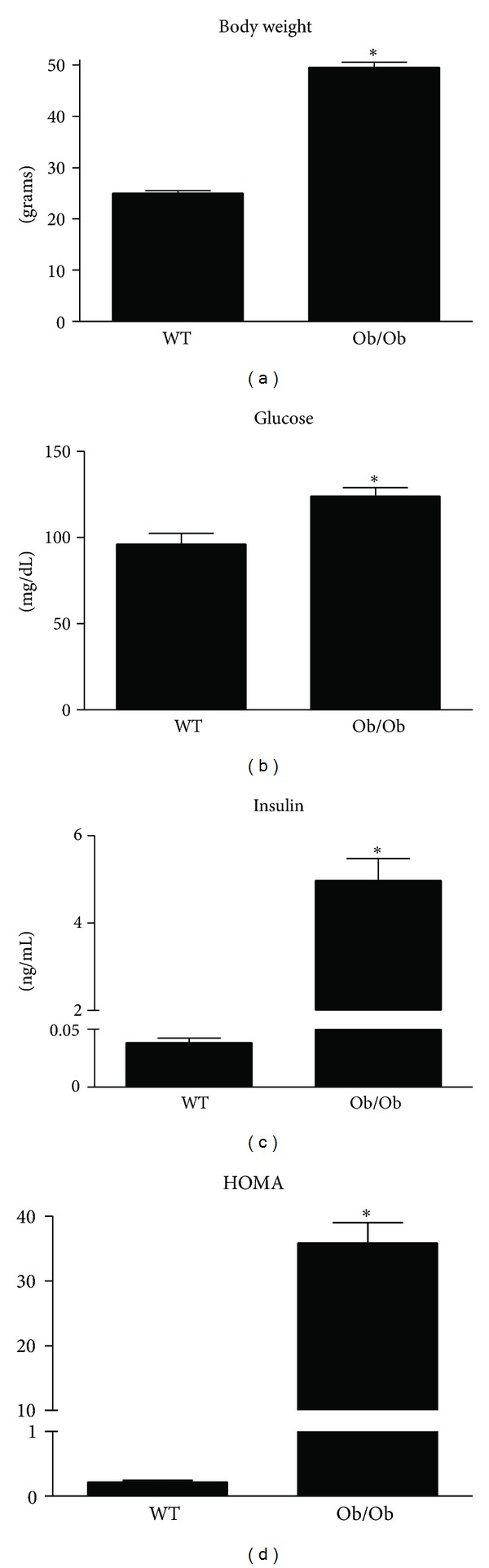
Increased body weight and reduced insulin sensitivity are characteristics of leptin deficient Ob/Ob mice. Body weight (a), fasting blood glucose (b), fasting plasma insulin (c), and homeostasis model assessment (HOMA) (d) were elevated in 14-week-old male Ob/Ob mice as compared to age-matched male wild-type (WT) mice. Values are means ± SE (*n* = 7); *P < 0.05.

Overnutrition is considered the most common cause of NAFLD, with an estimated incidence of 15–20% in Western populations [18]. The hyperphagic, leptin-deficient Ob/Ob mouse model is commonly used for the study of NAFLD, as it develops many of the metabolic abnormalities associated with human NAFLD including excess caloric consumption, obesity, insulin resistance, and dyslipidemia. In the current study, 14-week-old Ob/Ob mice were observed to have increased body weight as compared to age-matched wild-type (WT) mice (Figure 1), and this difference in body weight was due, in part, to increased fat pad mass (gonadal fat pad mass 3.1 g ± 0.1 versus 0.4 g ± 0.0; *P* < 0.05). In addition, Ob/Ob mice exhibited elevated fasting blood glucose and plasma insulin concentrations (Figure 1). Calculated HOMA indices demonstrated that Ob/Ob mice were insulin resistant as compared to WT controls (Figure 1). These data are consistent with previous studies of the Ob/Ob mouse and have been shown to be caused in part by hyperphagia due to a lack of leptin signaling [19, 20].

**Figure 2:**
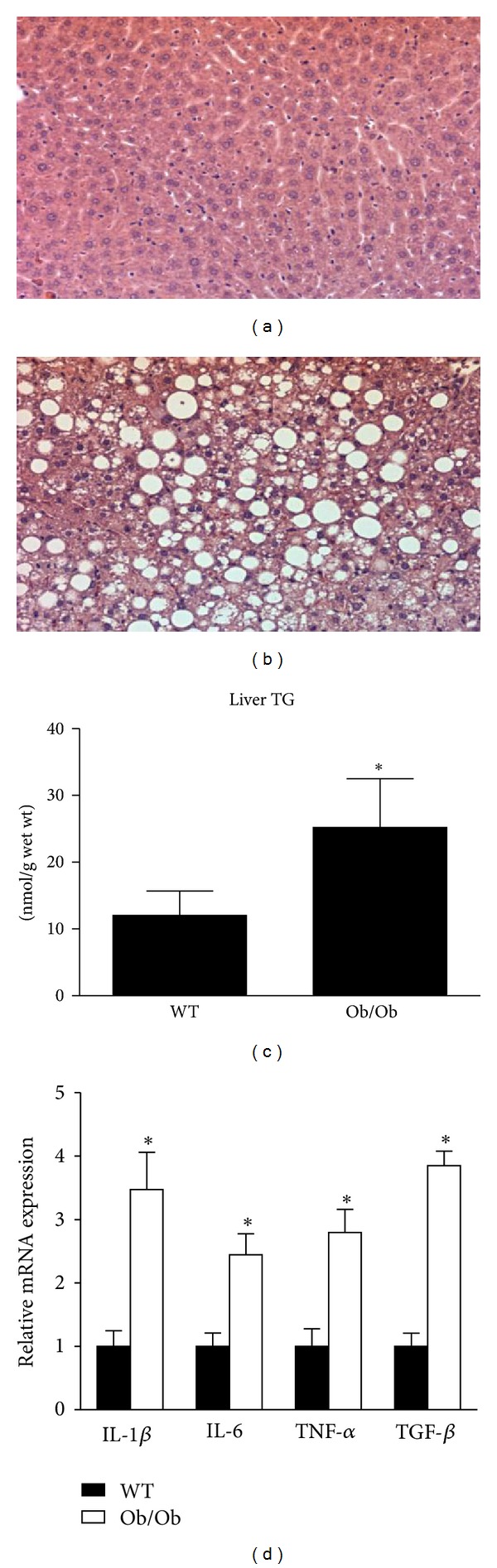
Ob/Ob mice display hepatic lipid accumulation and increased hepatic inflammation. Representative H&E stained images from the livers of age-matched wild-type (WT) (a) and Ob/Ob (b) mice fed a standard rodent chow. Triglyceride content (c) and relative gene expression of inflammatory factors (d) were significantly increased in the livers of Ob/Ob mice. Values are means ± SE (*n* = 7); *P < 0.05.

Livers excised from the Ob/Ob mice were significantly larger than those from WT animals (3.4 g ± 0.1 versus 0.8 g ± 0.0; *P* < 0.05). Histological sections revealed both micro- and macrovesicular steatosis in the livers of Ob/Ob mice (Figure 2). When quantified by organic extraction, the triglyceride content of Ob/Ob livers was twice that of the WT mice (Figure 2). We also examined the relative mRNA expression of inflammatory markers in these livers and determined that IL-1*β*, IL-6, TNF-*α*, and TGF-*β* were all at least 2-fold higher (*P* < 0.05) in the Ob/Ob mice compared with WT mice (Figure 2). These data demonstrate that the Ob/Ob mice developed NAFLD, consisting of both robust hepatic steatosis and inflammation and are consistent with a recent publication providing a thorough pathological characterization of livers in Ob/Ob mice [21]. These authors reported extensive lipid deposition in the liver accompanied by mild to moderate necroinflammation and fibrosis, which is consistent with the pathological manifestation of NAFLD in a large percentage of humans [21–23]. The clinical implications of these pathological changes to the liver include impaired insulin sensitivity. Ectopic lipid accumulation in the liver and elevations in inflammatory factors have been implicated in insulin resistance and may have contributed to the elevations in blood glucose and plasma insulin observed in the present study [3, 24, 25].

**Figure 3:**
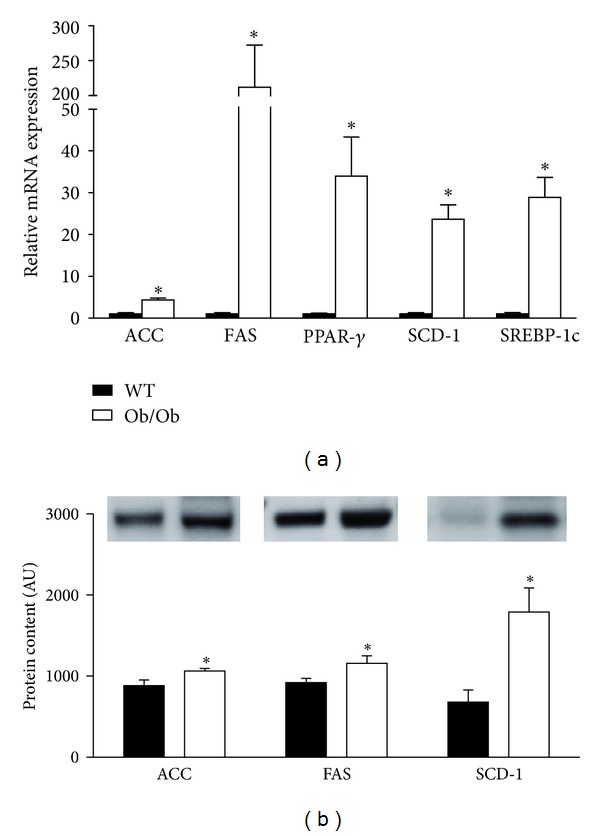
Markers of hepatic lipogenesis are elevated in Ob/Ob mice. Gene expression (a) and protein content (b) of hepatic lipogenesis markers were elevated in 14-week-old male Ob/Ob mice as compared to age-matched male wild-type (WT) mice. Values are means ± SE (*n* = 7); *P < 0.05.

We further investigated the observed increase in hepatic triglyceride content. Relative mRNA expression and protein content of key lipogenic factors were measured in the livers of Ob/Ob and WT mice. Consistent with our observation of increased lipid accumulation, mRNA expression of hepatic *de novo* lipogenic genes ACC, FAS, peroxisome proliferator-activated receptor (PPAR) **γ**, sterol regulatory element-binding protein-1c (SREBP-1c), and SCD-1 were dramatically elevated in the Ob/Ob mice (Figure 3). Similarly, the protein content of SCD-1, FAS, and ACC was more abundant in the livers of Ob/Ob mice (Figure 3). Consistent with these data, it has been previously shown in Ob/Ob mice that increased rates of hepatic fatty acid synthesis are associated with increased nuclear SREBP-1c protein and mRNA levels for known SREBP target genes involved in fatty acid biosynthesis [26]. Hepatic lipogenesis is upregulated in obese and insulin-resistant humans and has been shown to be a significant contributor towards NAFLD development in humans [27–29]. Conversely, moderate reductions in hepatic lipid content are associated with improvements in insulin sensitivity and therefore lipogenic signaling pathways continue to be explored as potential therapeutic targets [30–32].

**Figure 4:**
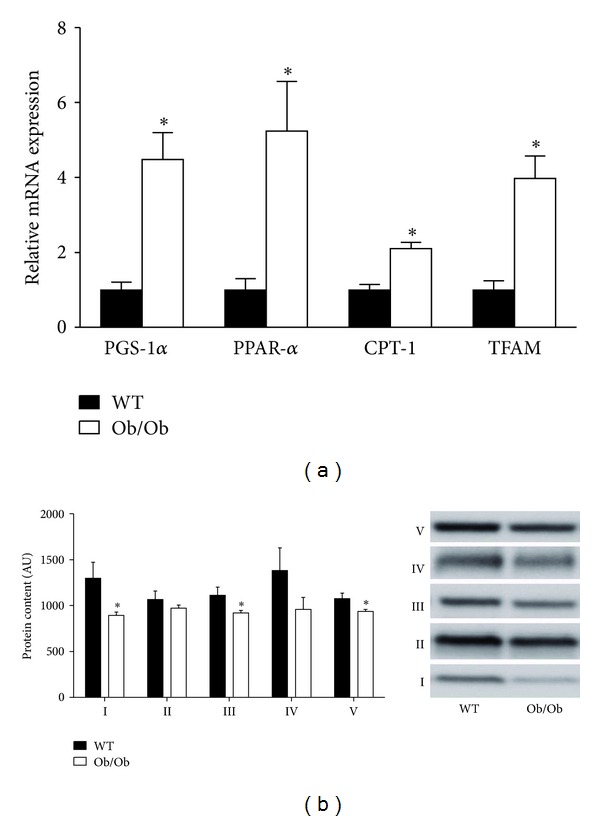
Ob/Ob mice exhibit increased hepatic mitochondrial gene expression but reduced hepatic mitochondrial protein content. Age-matched 14-week-old male Ob/Ob mice have increased gene expression of hepatic mitochondrial markers (a), while protein content of the hepatic mitochondrial oxidative phosphorylation complexes I to V of the electron transport chain (b) was reduced as compared to wild-type (WT) mice. Values are means ± SE (*n* = 7); *P < 0.05.

**Figure 5:**
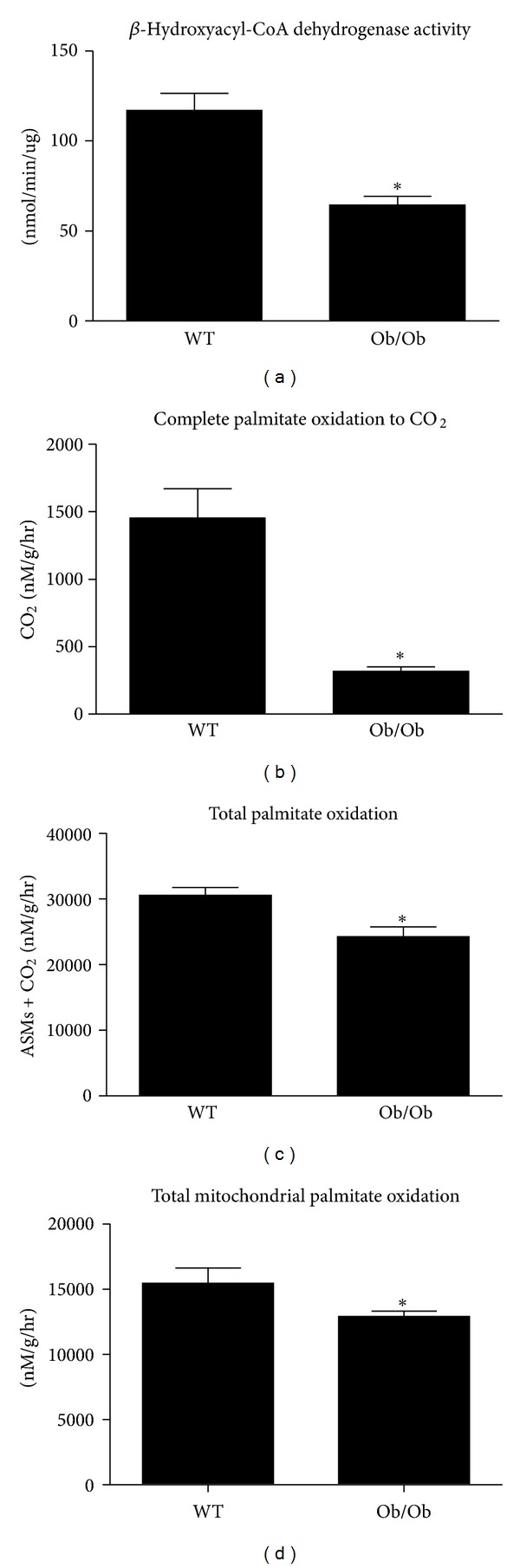
Hepatic mitochondrial function is reduced in Ob/Ob mice. Hepatic mitochondrial *β*-HAD (a), complete palmitate oxidation (b), and total palmitate oxidation (c) were determined in fresh liver homogenate preparations from age-matched wild-type (WT) and Ob/Ob mice. Values are means ± SE (*n* = 7); *P < 0.05.

Alterations in hepatic mitochondrial content and/or function also are known to contribute to hepatic lipid accumulation [7, 33]. Here we observed that genes involved in mitochondrial biogenesis (peroxisome proliferator-activated receptor *γ* coactivator- (PGC-)1*α*, mitochondrial transcription factor A, (TFAM)) and fatty acid oxidation (PPAR-*α*, carnitine palmitoyltransferase-1 (CPT-1)) were upregulated in the livers of Ob/Ob mice (Figure 4). However, hepatic markers of mitochondrial protein content (oxidative phosphorylation subunits I, III, and V) were significantly lower in Ob/Ob mice (Figure 4). Given the apparent discrepancy between gene expression and protein content of mitochondrial markers, we performed functional assays to further characterize hepatic mitochondria. Ob/Ob mice exhibited a ~50% reduction in hepatic *β*-hydroxyacyl-CoA dehydrogenase (*β*-HAD) activity, which is the rate-limiting step in mitochondrial *β*-oxidation (Figure 5). In addition, complete hepatic palmitate disposal (CO_2_ production) was reduced by 75% (*P* < 0.01), and total (CO_2_  + acid soluble metabolites) and mitochondria palmitate oxidation were reduced by ~25% (*P* < 0.05) in the livers of Ob/Ob mice compared with WT (Figure 5). 

Collectively, the findings of reduced hepatic mitochondrial protein content and function as well as an upregulation in hepatic *de novo* lipogenesis in the Ob/Ob mice are consistent with previous work examining these different pathways known to contribute to development of NAFLD induced by overnutrition [7, 14, 15]. Others have previously demonstrated that Ob/Ob mice have reduced hepatic mitochondrial respiratory chain activity [11] and increased in several key genes associated with *de novo* lipogenesis [12, 34, 35]. We extend these findings and highlight reduced hepatic oxidative phosphorylation proteins I, III, and V as well as dramatic reductions in complete and total palmitate oxidation in the Ob/Ob mice. Reductions in complete palmitate disposal leads to an accumulation of acetyl-CoA metabolites that can be exported as ketones or converted to acetyl-carnitines by the enzyme carnitine acetyltransferase and then accumulate in the cell cytosol or leak into plasma. Increased acetyl carnitine levels in muscle and plasma have been reported in various models of obesity [36, 37]. Moreover, previous reports indicate that the products of incomplete oxidation (acetyl-CoA and acetyl-carnitine) may also be directed to fatty acid biosynthesis pathways [38], likely tying together the oxidation and synthesis pathways in this animal model. 

## 4. Conclusions

The objective of the current study was to determine the underlying potential contribution of alterations in hepatic mitochondrial function and content as well as *de novo* lipogenesis to NAFLD development in the commonly studied model of obesity, the leptin-deficient Ob/Ob mouse. Our data show that despite significant elevations in hepatic mRNA expression for PGC-1*α*, PPAR-*α*, and TFAM, Ob/Ob mice exhibited significant impairments in hepatic mitochondrial function, including reduced *β*-HAD activity and fatty acid oxidation, as well as reduced mitochondrial electron transport chain proteins. Moreover, the Ob/Ob mice also demonstrate a dramatic upregulation in markers of hepatic *de novo* lipogenesis compared with nonhyperphagic WT mice. Collectively, these findings suggest that hepatic mitochondrial dysfunction and/or a reduction in mitochondrial content along with an upregulation in *de novo* lipogenesis contributes to obesity-associated NAFLD in this model. Future longitudinal studies will be required to help elucidate the timing of hepatic mitochondrial dysfunction and lipogenesis in relation to NAFLD development. This additional understanding will assist in selecting therapeutic targets for altering hepatic lipid metabolism for the prevention and/or treatment of NAFLD.

## Figures and Tables

**Table 1:** qRT-PCR primer sequences.

Gene	Forward sequence	Reverse sequence
ACC	AACATCCCCACGCTAAACAG	CTGACAAGGTGGCGTGAAG
CPT-1	AGTGGCCTCACAGACTCCAG	GCCATGTTGTACAGCTTCC
Cyclophilin	ATGTGGTTTTCGGCAAAGTT	TGACATCCTTCAGTGGCTTG
FAS	CCCTTGATGAAGAGGGATCA	GAACAAGGCGTTAGGGTTGA
IL-1*β*	TCACAGCAGCACATCAACAA	TGTCCTCATCCTGGAAGGTC
IL-6	ACCAGAGGAAATTTTCAATAGGC	TGATGCACTTGCAGAAAACA
PGC-1*α*	ATGTGTCGCCTTCTTGCTCT	ATCTACTGCCTGGGGACCTT
PPAR-*α*	CAGTGGGGAGAGAGGACAGA	AGTTCGGGAACAAGACGTTG
PPAR-*γ*	GATGGAAGACCACTCGCATT	AACCATTGGGTCAGCTCTTG
SCD-1	GCTGGGCAGGAACTAGTGAG	GAAGGCATGGAAGGTTCAAA
SREBP-1c	ATCTCCTAGAGCGAGCGTTG	TATTTAGCAACTGCAGATATCCAAG
TFAM	TCCAAGCCTCATTTACAAGC	CCAAAAAGACCTCGTTCAGC
TGF-*β*	AAGTTGGCATGGTAGCCCTT	GCCCTGGATACCAACTATTGC
TNF-*α*	ATGGGCTTTCCGAATTCAC	GAGGCAACCTGACCACTCTC
